# Supplementary figures and images for: Preoperative diagnoses and identification rates of unexpected gallbladder cancer
Source: PLoS One. 2020 Sep 18;15(9):e0239178. doi: 10.1371/journal.pone.0239178 (PMC7500683; doi:10.1371/journal.pone.0239178)

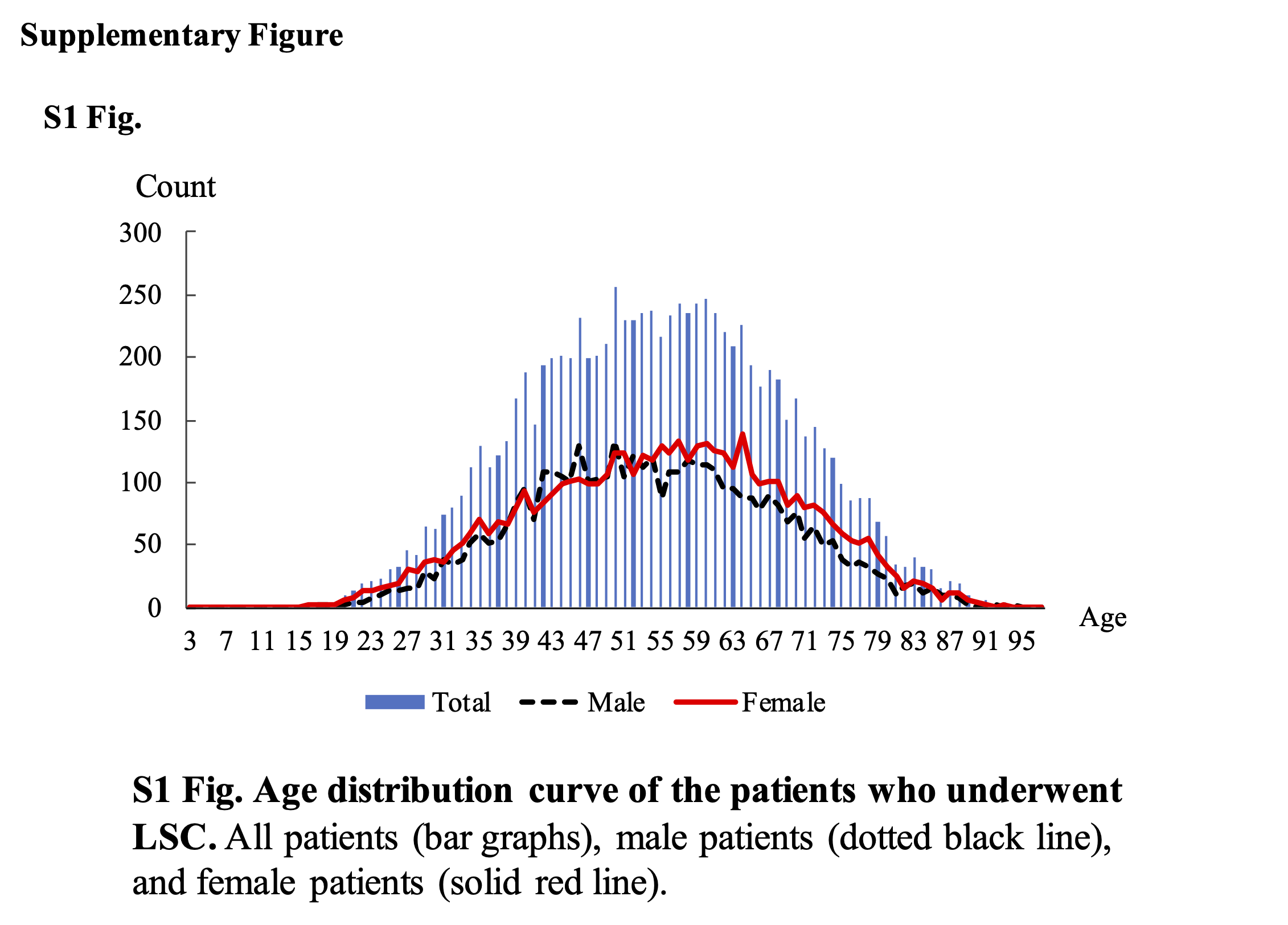

Supplement: S1 Fig — All patients (bar graphs), male patients (dotted black line), and female patients (solid red line). (TIFF) [file pone.0239178.s001.tiff]
