# Supplementary material for: Preoperative diagnoses and identification rates of unexpected gallbladder cancer
Source: PLoS One. 2020 Sep 18;15(9):e0239178. doi: 10.1371/journal.pone.0239178 (PMC7500683; doi:10.1371/journal.pone.0239178)
Supplement: S1 Table — (DOCX) [file pone.0239178.s002.docx]

**S1 Table. Patients’ demographics.**

|  | Patient numbers |
| --- | --- |
| Total | 9200 |
| Sex |  |
| Male | 4291 |
| Female | 4909 |
| Age, years (median) | 3–97 (55) |
| Final diagnoses |  |
| Cholecystolithiasis and choledocholithiasis | 5582 |
| Chronic cholecystitis/cholecystitis | 1355 |
| Acute cholecystitis | 956 |
| Benign tumor (including gallbladder polyp) | 639 |
| Adenomyomatosis | 359 |
| Gallbladder cancer | 77 |
| Other (Biliary dyskinesia, Congenital biliary dilatation) | 9 |
| Unknown | 223 |
