# Supplementary material for: Preoperative diagnoses and identification rates of unexpected gallbladder cancer
Source: PLoS One. 2020 Sep 18;15(9):e0239178. doi: 10.1371/journal.pone.0239178 (PMC7500683; doi:10.1371/journal.pone.0239178)
Supplement: S2 Table — (DOCX) [file pone.0239178.s003.docx]

**S2 Table. Numbers of patients with multiple diagnostic terms.**

| Diagnostic terms | Category | N |
| --- | --- | --- |
| Cholecystolithiasis and adenomyomatosis | Cholecystolithiasis | 559 |
| Cholecystolithiasis and choledocholithiasis | Cholecystolithiasis | 133 |
| Cholecystolithiasis and gallbladder polyp | Cholecystolithiasis | 98 |
| Cholecystolithiasis, gallbladder polyp, and adenomyomatosis | Cholecystolithiasis | 16 |
| Chronic cholecystitis and adenomyomatosis | Chronic cholecystitis/ cholecystitis | 124 |
| Chronic cholecystitis and choledocholithiasis | Chronic cholecystitis/ cholecystitis | 43 |
| Chronic cholecystitis and gallbladder polyp | Chronic cholecystitis/ cholecystitis | 21 |
| Acute cholecystitis and choledocholithiasis | Acute cholecystitis | 66 |
| Acute cholecystitis and adenomyomatosis | Acute cholecystitis | 13 |
| Gallbladder polyp and adenomyomatosis | Benign tumor | 33 |
| Gall bladder polyp and cholecystolithiasis | Benign tumor | 24 |
| Other (less than 10 patients) |  | 109 |
| Total |  | 1239 |
