# Supplementary material for: Preoperative diagnoses and identification rates of unexpected gallbladder cancer
Source: PLoS One. 2020 Sep 18;15(9):e0239178. doi: 10.1371/journal.pone.0239178 (PMC7500683; doi:10.1371/journal.pone.0239178)
Supplement: S3 Table — (DOCX) [file pone.0239178.s004.docx]

**S3 Table. Preoperative diagnoses of the 77 patients with gallbladder cancer.**

| Preoperative diagnosis | N (%) |
| --- | --- |
| Chronic cholecystitis | 28 (36.4%) |
| Benign tumor | 16 (20.8%) |
| Suspected gallbladder cancer | 14 (18.2%) |
| Acute cholecystitis | 13 (16.9%) |
| Adenomyomatosis | 3 (3.9%) |
| Cholecystolithiasis | 3 (3.9%) |
| Total | 77 |
