# Supplementary material for: Preoperative diagnoses and identification rates of unexpected gallbladder cancer
Source: PLoS One. 2020 Sep 18;15(9):e0239178. doi: 10.1371/journal.pone.0239178 (PMC7500683; doi:10.1371/journal.pone.0239178)
Supplement: S4 Table — (DOCX) [file pone.0239178.s005.docx]

**S4 Table. Pre/postoperative findings for the patients undergoing laparoscopic cholecystectomy (LSC), categorized according to the final diagnoses.**

|  |  | Gallbladder cancer | Cholecystolithiasis and choledocholithiasis | Chronic cholecystitis/ cholecystitis | Acute cholecystitis | Benign tumor | Adenomyomatosis | Other | Unknown | Total |
| --- | --- | --- | --- | --- | --- | --- | --- | --- | --- | --- |
| Variable |  | 77 | 5582 | 1355 | 956 | 639 | 359 | 9 | 223 | 9200 |
| Age | <60 | 15 (19.5%) | 3492 (62.6%) | 800 (59.0%) | 499 (52.2%) | 498 (77.9%) | 283 (78.8%) | 8 (88.9%) | 139 (62.3%) | 5734 (62.3%) |
|  | ≧60 | 62 (80.5%) | 2090 (37.4%) | 555 (41.0%) | 457 (47.8%) | 141 (22.1%) | 76 (21.2%) | 1 (11.1%) | 84 (37.7%) | 3466 (37.7%) |
| Sex | Male | 36 (46.8%) | 2340 (41.9%) | 687 (50.7%) | 597 (62.4%) | 361 (56.5%) | 173 (48.2%) | 2 (22.2%) | 95 (42.6%) | 4291 (46.6%) |
|  | Female | 41 (53.2%) | 3242 (58.1%) | 668 (49.3%) | 359 (37.6%) | 278 (43.5%) | 186 (51.8%) | 7 (77.8%) | 128 (57.4%) | 4909 (53.4%) |
| Gallbladder imaging on DIC-CT | |  |  |  |  |  |  |  |  |  |
|  | Gallbladder- negative contrast | 19 (48.7%) | 1316 (31.6%) | 352 (36.0%) | 423 (83.3%) | 14 (4.8%) | 22 (20.8%) | 0 (0%) | 8 (30.8%) | 2154 (35.3%) |
|  | Positive contrast | 20 (51.3%) | 2838 (68.4%) | 627 (64.0%) | 85 (16.7%) | 280 (95.2%) | 84 (79.2%) | 3 (100%) | 18 (69.2%) | 3955 (64.7%) |
| Thickened wall | |  |  |  |  |  |  |  |  |  |
|  | positive | 51 (76.1%) | 2979 (58.8%) | 986 (73.3%) | 864 (95.5%) | 163 (27.9%) | 293 (87.5%) | 3 (42.9%) | 25 (53.2%) | 5364 (64.2%) |
|  | negative | 16 (23.9%) | 2091 (41.2%) | 359 (26.7%) | 41 (4.5%) | 421 (72.1%) | 42 (12.5%) | 4 (57.1%) | 22 (46.8%) | 2996 (35.8%) |

DIC-CT, drip infusion cholangiography with computed tomography
