# Supplementary material for: Preoperative diagnoses and identification rates of unexpected gallbladder cancer
Source: PLoS One. 2020 Sep 18;15(9):e0239178. doi: 10.1371/journal.pone.0239178 (PMC7500683; doi:10.1371/journal.pone.0239178)
Supplement: S5 Table — (DOCX) [file pone.0239178.s006.docx]

**S5 Table. Pre/postoperative findings for gallbladder cancer patients categorized according to the preoperative diagnoses.**

|  |  | Suspected gallbladder cancer | Cholecystolithiasis and choledocholithiasis | Chronic cholecystitis/ cholecystitis | Acute cholecystitis | Benign tumor | Adenomyomatosis | Total |
| --- | --- | --- | --- | --- | --- | --- | --- | --- |
| Variable |  | 14 | 3 | 28 | 13 | 16 | 3 | 77 |
| Age | <60 | 3 (21.4%) | 1 (33.3%) | 5 (17.9%) | 1 (7.7%) | 5 (31.3%) | 0 (0%) | 15 (19.5%) |
|  | ≧60 | 11 (78.6%) | 2 (66.7%) | 23 (82.1%) | 12 (92.3%) | 11 (68.7%) | 3 (100%) | 62 (80.5%) |
| Sex | Male | 5 (35.7%) | 1 (33.3%) | 16 (57.1%) | 5 (38.5%) | 8 (50.0%) | 1 (33.3%) | 36 (46.8%) |
|  | Female | 9 (64.3%) | 2 (66.7%) | 12 (42.9%) | 8 (61.5%) | 8 (50.0%) | 2 (66.7%) | 41 (53.2%) |
| Gallbladder imaging on DIC-CT | |  |  |  |  |  |  |  |
|  | Gallbladder- negative contrast | 4 (50.0%) | 1 (100%) | 8 (57.1%) | 0 (0%) | 5 (71.4%) | 2 (66.7%) | 20 (51.3%) |
|  | Positive contrast | 4 (50.0%) | 0 (0%) | 6 (42.9%) | 6 (100%) | 2 (28.6%) | 1 (33.3%) | 19 (48.7%) |
| Thickened wall | |  |  |  |  |  |  |  |
|  | positive | 8 (72.7%) | 3 (100%) | 16 (64.0%) | 12 (100%) | 9 (69.2%) | 3 (100%) | 51 (76.1%) |
|  | negative | 3 (22.3%) | 0 (0%) | 9 (36.0%) | 0 (0%) | 4 (30.8%) | 0 (0%) | 16 (23.9%) |

DIC-CT, drip infusion cholangiography with computed tomography
