# Supplementary material for: Preoperative diagnoses and identification rates of unexpected gallbladder cancer
Source: PLoS One. 2020 Sep 18;15(9):e0239178. doi: 10.1371/journal.pone.0239178 (PMC7500683; doi:10.1371/journal.pone.0239178)
Supplement: S6 Table — (DOCX) [file pone.0239178.s007.docx]

**S6 Table. Comparison of pre/postoperative findings between the patients finally diagnosed as having unexpected gallbladder cancer (UGBC) with the patients finally diagnosed as having benign disease (sex and gallbladder imaging on drip infusion cholangiography with computed tomography (DIC-CT).**

|  |  | Female patients | p-value | DIC-CT negative contrast | p-value |
| --- | --- | --- | --- | --- | --- |
| Cholecystolithiasis and choledocholithiasis | UGBC | 2/3 (66.7%)* | 0.76 | 0/1 (0%) | 0.50 |
|  | Benign | 3242/5582 (58.1%) |  | 1316 /4154 (31.6%) |  |
| Chronic cholecystitis/ cholecystitis | UGBC | 12/28 (42.9%) | 0.50 | 6/14 (42.9%) | 0.59 |
|  | Benign | 668/1355 (49.3%) |  | 352/979 (36.0%) |  |
| Acute cholecystitis | UGBC | 8/13 (61.5%) | 0.077 | 6/6 (100%) | 0.27 |
|  | Benign | 359/956 (37.6%) |  | 423/508 (83.3%) |  |
| Benign tumor | UGBC | 8/16 (50.0%) | 0.60 | 2/7 (28.6%) | 0.006 |
|  | Benign | 278/639 (43.5%) |  | 14/294 (4.8%) |  |
| Adenomyomatosis | UGBC | 2/3 (66.7%) | 0.61 | 1/3 (33.3%) | 0.60 |
|  | Benign | 186/359 (51.8%) |  | 22/106 (20.8%) |  |

*Data indicate the number of positive cases/number of total cases (%).
