# Supplementary material for: Preoperative diagnoses and identification rates of unexpected gallbladder cancer
Source: PLoS One. 2020 Sep 18;15(9):e0239178. doi: 10.1371/journal.pone.0239178 (PMC7500683; doi:10.1371/journal.pone.0239178)
Supplement: S7 Table — (DOCX) [file pone.0239178.s008.docx]

**S7 Table. Identification rates of incidental pathologically-detected gallbladder cancer cases categorized according to the preoperative diagnoses.**

| Preoperative diagnosis | Incidental pathologically-detected gallbladder cancer number/total count (percentages) |
| --- | --- |
| Chronic cholecystitis/ cholecystitis | 22/1377 (1.6%) |
| Benign tumor | 6/645 (0.93%) |
| Acute cholecystitis | 6/962 (0.62%) |
| Adenomyomatosis | 3/362 (0.83%) |
| Cholecystolithiasis and choledocholithiasis | 2/5584 (0.036%) |
| Total | 39/9162 (0.43%) |
